# Some Cases of Arm Presentation

**Published:** 1878-01

**Authors:** 


Some Cases of Arm Presentation.—*Case* 1.—Mrs. B., a stout, healthy woman of middle age, was taken in labor June 18th, 1877. She had always had great difficulty in her confinements, and, with but one exception, had lost her children in the birth. The living one died in infancy.

When seen in the forenoon, she was having strong pains at short intervals. Upon examination, the mouth of the womb was found largely dilated, and the bag of waters tensely protruding. The pressure of the examining finger, though slight, caused the rupture of the bag, and an arm was found to be the presenting part. She was put upon her elbows and knees, and the child was turned with very little trouble. The body was extracted with considerable difficulty, but the head could not be drawn down, notwithstanding the application of the greatest force consistent with safety. Unfortunately, I had not brought my long forceps with me, and the small pair of short ones I had in my obstetrical bag were of no use. The time lost in getting my long forceps—about half an hour—was fatal to the child, of course. They were easily applied, and delivery effected in a very few minutes. The placenta was immediately removed, and there was no hemorrhage. Recovery was rapid, without a bad symptom. The cause of difficulty in this case was a narrow superior strait—autero-posteriorly from a projecting sacral promontory—which opposed such resistance to the passage of the head, that it was feared craniotomy would be necessary; but with the leverage and power of the long forceps, this was obviated. The knee-elbow position rendered version a much less difficult operation than I had previously found it in the dorsal position, and but for the delay in the delivery of the head, the child would have been saved. This shows the necessity of being always provided with a pair of long forceps. And I had made it a rule to always carry them with me when called to a case of labor; but not having had any use for them for some time, I had contented myself with carrying a very lfght and short pair.

*Case* 2.—Mrs. C., a small, well-made, healthy, hard-working Irish woman, had been treated by me, for prolapse of the womb, in 1874. The vagina was so relaxed she could only get relief by wearing a large Physick's globe pessary, which answered perfectly. To my surprise, I was called to attend her in confinement in February, 1875. Not suspecting anything wrong, I waited patiently for nature to do her share of the work, but the labor seemed to make no progress, and time wore on slowly. As soon as the womb had opened sufficiently to make a satisfactory examination, it was discovered, as feared from the unusual duration of the first stage, that it was a "cross birth"—an arm presentation. Delivery was effected with much difficulty by podalic version; child born dead. The woman made a good recovery. In this case version was performed in the dorsal position.

On the 11th of August, 1877, I was again called to attend her in her confinement. As on the last occasion, the first stage was very slow. Feeling uneasy on account of the trouble I had had with her before, I soon discovered that all was not right, and to my horror, found it to be another arm presentation. The head was resting in the left iliac fossa, and as no outline of the child's body could be felt, it was inferred to be the left shoulder and arm. My unfortunate experience made me loth to resort to version. Thinking the head might be forced down into the pelvis, this was tried by external manipulation; but it would not move. Conjoined external and internal manipulation was tried; but to make matters worse, the cord prolapsed, and no progress was made. She was then put upon her knees and elbows, notwithstanding she declared I was going to kill her "intirely;" and while in this position the cord was replaced, the arm and shoulder pressed up, and the head forced down by the other hand outside until a strong pain came on, when she was quickly placed on her back. Everything was found to be right. The head had engaged, and there was no chance for the cord to come down any more. She was safe, and I was immensely relieved. Strong, steady pains replaced the unsatisfactory ones that had been going on so long, and the labor progressed naturally. In a short time it was over, and a *live* male child was born, which, by the vigor of his movements and his cries, showed he was "all right."

I had been filled with anxiety lest the child should be born dead, on account of the prolapse of the cord; for it was not pulsating when down, and although replaced quickly, I was not sure that the circulation was still going on through it. But as there had been no hemorrhage indicative of any separation of the placenta, it was hoped and believed the child's vitality was preserved. Here, again, the knee-elbow position was of the greatest advantage, rendering the restoration of the prolapsed cord easy, and enabling me to disengage the presenting arm from its position and to press the head down into the superior strait, where, having once engaged, the labor was suddenly changed from the most difficult and dangerous to a safe and easy one.

A consideration of the mechanics of the knee-elbow position will show how it renders easy and possible obstetric operations that are otherwise difficult or impossible. In it the womb and its contents are passive, and free from all pressure from the superincumbent viscera and the action of the abdominal muscles, so that motion can be imparted to the child in utero, unopposed by the resistance of their compressing force.

In addition to the three cases mentioned, it has fallen to my lot to have had two more cases of arm presentation, making five in all, and the trouble and anxiety they gave me impels me to pay my mite of tribute to so invaluable a resource as the knee-elbow position for rectifying the presentation and rendering delivery safe and easy.—*New Orleans Medical and Surgical Journal.*

